# Revision Arthroscopic Bankart Repair for Anterior Shoulder Instability After a Failed Arthroscopic Soft-Tissue Repair Yields Comparable Failure Rates to Primary Bankart Repair: A Systematic Review

**DOI:** 10.1177/15563316211030606

**Published:** 2021-07-23

**Authors:** Ajaykumar Shanmugaraj, Seaher Sakha, Tushar Tejpal, Timothy Leroux, Jacob M Kirsch, Moin Khan

**Affiliations:** 1 Division of Orthopaedic Surgery, Department of Surgery, McMaster University, Hamilton, ON, Canada; 2 Department of Surgery, University of Toronto, Toronto, ON, Canada; 3 Department of Orthopaedic Surgery, New England Baptist Hospital, Boston, MA, USA

**Keywords:** revision, Bankart repair, soft tissue, shoulder, instability

## Abstract

**Background:**

The management of recurrent instability after arthroscopic Bankart repair remains challenging. Of the various treatment options, arthroscopic revision repairs are of increasing interest due to improved visualization of pathology and advancements in arthroscopic techniques and instrumentation.

**Purpose:**

We sought to assess the indications, techniques, outcomes, and complications for patients undergoing revision arthroscopic Bankart repair after a failed index arthroscopic soft-tissue stabilization for anterior shoulder instability.

**Methods:**

We performed a systematic review of studies identified by a search of Medline, Embase, and PubMed. Our search range was from data inception to April 29, 2020. Outcomes include clinical outcomes and rates of complication and revision. The Methodological Index for Non-randomized Studies (MINORS) was used to assess study quality. Data are presented descriptively.

**Results:**

Twelve studies were identified, comprising 279 patients (281 shoulders) with a mean age of 26.1 ± 3.8 years and a mean follow-up of 55.7 ± 24.3 months. Patients had improvements in postoperative outcomes (eg, pain and function). The overall complication rate was 29.5%, the most common being recurrent instability (19.9%).

**Conclusion:**

With significant improvements postoperatively and comparable recurrent instability rates, there exists a potential role in the use of revision arthroscopic Bankart repair where the glenoid bone loss is less than 20%. Clinicians should consider patient history and imaging findings to determine whether a more rigorous stabilization procedure is warranted. Large prospective cohorts with long-term follow-up and improved documentation are required to determine more accurate failure rates.

## Introduction

Arthroscopic Bankart repair is commonly performed for shoulder instability and reported failure rates (ie, recurrent instability) range from 5% to 15% [4,23]. Various factors may contribute to recurrent instability following arthroscopic anterior stabilization, including presence and degree of bony glenoid defects, size and location of Hill-Sachs lesions, and generalized ligamentous laxity [4,30,31,39,45,47,51].

There are various treatment options for patients with recurrent instability after a primary Bankart repair [10]. Open procedures were once considered the gold standard for revision procedures however, reported failure rates range from 8% to 39% [1,10,15,22,30,32,39,41,49,51]. Arthroscopic revision repairs are of increasing interest due to improved visualization of pathology as well as advancements in arthroscopic techniques and instrumentation [5,13,25]. This approach is associated with reduced morbidity, early functional rehabilitation, and improved range of motion [23,37].

Currently, there is extensive literature reporting the effectiveness of revision anterior shoulder stabilization techniques, particularly open revision Bankart/Latarjet repair following failed arthroscopic Bankart procedures [9,14,28,36]. There has also been increasing interest in the use of an arthroscopic Bankart repair in the revision setting in mixed populations (ie, failed open or arthroscopic index procedure) [1,50]. However, there exists a dearth of evidence on arthroscopic revision Bankart procedures for recurrent anterior shoulder instability following a failed primary arthroscopic Bankart repair.

The purpose of this review was to assess the indications, techniques, outcomes, and complications for patients undergoing revision arthroscopic Bankart repair after a failed index arthroscopic soft-tissue stabilization. It was hypothesized that patients with minimal glenoid bone loss would achieve moderate to excellent postoperative outcomes with a low to moderate complication rate that is comparable to primary arthroscopic Bankart repair. We also predicted that there would be a limited quantity and quality of available evidence.

## Methods

### Search Strategy

PubMed, Embase, and Medline were searched for studies on revision arthroscopic Bankart repair after a failed index arthroscopic soft-tissue repair from data inception to April 29, 2020. The search terms included “shoulder,” “Bankart repair,” “revision,” “anterior,” and similar phrases (Table 1). To ensure that articles were not missed, the search terms were entered onto Google Scholar and ClinicalTrials.gov. The research question and inclusion and exclusion criteria were established a priori. Conference proceedings, articles published ahead of print, and gray literature were searched. Inclusion criteria were (1) arthroscopic revision shoulder surgery, (2) anterior instability, (3) arthroscopic soft-tissue index procedure, (4) at least 1 outcome reported and stratified for population of interest, (5) human studies, and (6) English language. The exclusion criteria were (1) multidirectional instability, (2) any other major shoulder procedure (eg, glenoid reconstructions, bony procedures) supplemented with arthroscopic surgery, (3) open index procedure (eg, open Bankart, Latarjet, modified Bristow, etc.), (4) review articles, (5) non-surgical treatment studies (eg, conservative treatment, technique articles without outcomes, etc.), (6) cadaver/non-human studies, and (7) case reports.

**Table 1.** Study characteristics.

Author	Study design (level of evidence)	Total sample size	Study group	% Male	Mean age (SD or range)	Mean follow-up (range)	Post-Operative Recurrent Instability	MINORS Consensus Score^a^	Limitations
Elamo et al [12]	Retrospective Cohort (III)	48	30	81	31.9	93.6	13/30	15/24	-Retrospective
									-Small sample size
									-High drop-out rate
Gill et al [18]	Retrospective Cohort (III)	43	16	NR	NR	60 (24-84)	0/16	7/16	-Retrospective
									- Limited reporting of data
Slaven et al [42]	Case Series	53	53	NR	22.9 ± 4.3	73.2 (4.8-154.8)	19/53	8/16	- Retrospective
									- Limited reporting of data
Frank et al [16]	Retrospective Cohort (III)	91	62 (63 shoulders)	74	23.2 ± 6.9	46.9 ± 16.8	12/63	11/24	- Retrospective
									- Limited reporting of data
Stein et al [44]	Retrospective Cohort (III)	69	23	82.8	31.8 ± 10.8	28.0 ± 15.6	1/23	18/24	- Retrospective
									- Small sample size
Imhoff et al [20]	Retrospective Cohort (III)	221	7	73.7^b^	28.0 (14.4-59.2)^b^	37.4 ± 15.8^b^	0/7	17/24	- Retrospective
									- Large overall sample size, but small sample size for population of interest
									- Limited reporting of data
Barnes et al [2]	Retrospective Cohort (III)	17 (18 shoulders)	9 (10 shoulders)	77.8	30 ± 13.6 (17-55)	40.9 ± 15.1	0/10	12/16	- Retrospective
									- Small sample size
Neri et al [35]	Retrospective Cohort (III)	12	6	83.3^b^	28 (18-56)^b^	34.4 (25-56)	2/6	11/16	- Retrospective
									- Small sample size
									- Poor documentation of data based off prior index procedure
Buckup et al [6]	Case Series (IV)	20	20	100	27.75 ± 7.19	28.7 ± 8.45	3/20	11/16	- Retrospective
									- Small sample size
Bartl et al [3]	Case Series (IV)	56	32	80^b^	29.4 (18-51) ^b^	37 (25-72) ^b^	4/32	12/16	- Poor documentation of data based off prior index procedure
Ryu and Ryu [40]	Case Series (IV)	15	11	90.9	23.9 ± 5.6 (17-35)	21.3 ± 9.6 (12-47)	1/11	9/16	- Retrospective
									- Small sample size
									- Limited reporting of data
Franceschi et al [15]	Case Series (IV)	10	10	80	25.6 (18-41)	68 (46-83)	1/10	13/16	- Retrospective
									- Small sample size

*MINORS* Methodological Index for Non-Randomized Studies, *NR* Not Reported.

a MINORS: 0-16 for non-comparative studies, 0-24 for comparative studies.

b Data not stratified for population of interest.

A systematic screening approach in accordance with Preferred Reporting Items for Systematic Reviews and Meta-analyses (PRISMA) [34] and Revised Assessment of Multiple Systematic Reviews (R-AMSTAR) [26] guidelines were employed from title to full text screening stages in duplicate by 2 independent reviewers (S.S., T.T.). Discrepancies were discussed and resolved with input by a third reviewer (A.S.). The references of included studies were also screened using the same systematic approach to capture any additional relevant articles.

### Quality Assessment

Using the *Journal of Bone & Joint Surgery* (JBJS) classification system for literature in the field of orthopedics, the level of evidence (I to IV) for each study was determined by the 2 reviewers independently and in duplicate [48]. The methodological quality of non-randomized comparative studies was evaluated using the methodological index for non-randomized studies (MINORS) [43]. A score of 0, 1, or 2 is given for each of the 12 items on the MINORS checklist with a maximum score of 16 for non-comparative studies and 24 for comparative studies. Methodological quality was categorized a priori as follows: a score of 0-8 or 0-2 was considered poor quality, 9-12 or 13-18 was considered fair quality, and 13-16 or 19-24 was considered excellent quality, for non-comparative and comparative studies, respectively.

Two reviewers (S.S., T.T.) independently abstracted relevant data from included articles and recorded the data onto a Google spreadsheet designed a priori. Demographic data included author, year of publication, sample size, study design and location, level of evidence, and patient demographics (eg, gender, age, etc.). Information regarding rehabilitation protocols and postoperative outcomes (surgical and radiographic) and complications was documented.

### Statistical Analysis

Due to high statistical and methodological heterogeneity, a meta-analysis could not be performed, and the results are summarized descriptively. Descriptive statistics such as mean, range, and measures of variance (eg, standard deviations, 95% confidence intervals [CI]) are presented where applicable. The intraclass correlation coefficient (ICC) was used to evaluate inter-reviewer agreement for assessing study quality. A kappa (κ) statistic was used to evaluate inter-reviewer agreement at all screening stages. Agreement was categorized a priori as follows: ICC/κ of 0.81 to 0.99 was considered as almost perfect agreement; ICC/κ of 0.61 to 0.80 was substantial agreement; ICC/κ of 0.41 to 0.60 was moderate agreement; 0.21 to 0.40 fair agreement and a ICC/κ value of 0.20 or less was considered slight agreement [27].

## Results

**Fig. 1.**
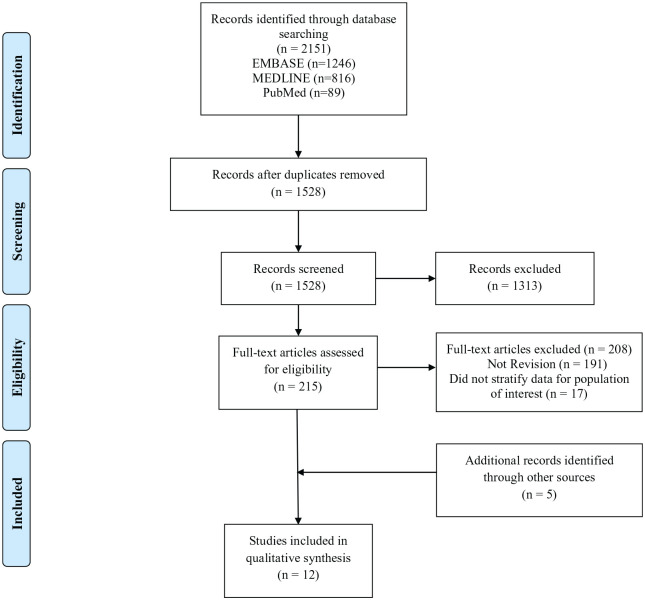
PRISMA flow diagram. *PRISMA* Preferred Reporting Items for Systematic Reviews and Meta-analyses.

The initial search yielded a total of 2151 articles. After excluding 623 duplicates, a systematic screening process found 12 articles that met inclusion criteria (Fig. 1). Of these, 5 were identified by reviewing reference lists of included studies or by a manual search through Google Scholar. Of the included studies, there were 7 retrospective cohort and 5 case series. Two of the included studies were conference abstracts (Table 1) [16,18].

The majority of studies in this systematic review were of Level III evidence (n = 7; 58.3%; Table 1). There was substantial agreement between reviewers at the title/abstract (κ = 0.74; 95% CI, 0.69-0.80) and full-text (κ = 0.70; 95% CI, 0.57-0.83) screening stages. There was substantial agreement for quality assessment scores (ICC = 0.99; 95% CI, 0.98-1.00). The mean MINORS scores for non-comparative and comparative studies were 10.4 ± 2.1 and 15.3 ± 3.1 respectively, which indicates fair quality of evidence for non-randomized studies (Table 1). The areas of best performance based on the MINORS checklist were an appropriate follow-up (N = 11; 100%), a clearly stated aim (N = 11; 91.7%), and endpoints appropriate for aim (N = 8; 72.7%). The area of worst performance was unbiased assessment of endpoints, which was not found in any of the included studies.

A total of 279 patients (281 shoulders) underwent revision arthroscopic Bankart repair for anterior instability after a failed arthroscopic soft-tissue stabilization. The mean sample size of patients undergoing revision arthroscopic Bankart repair after failed index arthroscopic stabilization per included study was 23.3 (range: 6-62). Of the included participants, 48.7% were male, with a mean age of 26.1 ± 3.8 years and a mean follow-up of 55.7 ± 24.3 months. Of the included studies, 5 did not specify sex distribution or mean age [3,18,20,35,42], and 3 did not specify mean follow-up for the population of interest [3,20,35] (Table 1).

The overall complication rate in our systematic review was 29.5% (n = 83). The most frequent complication was recurrent instability (19.9%; N = 56). Recurrent instability was defined as a postoperative dislocation in 4 studies [15,35,40,44], a traumatic event requiring surgical intervention in 2 studies [6,12], and a subluxation or dislocation in 2 studies [3,16]. Other common complications included osteoarthritis (6.8%; N =19), persistent postoperative apprehension (2.5%; N = 7), and traumatic fracture of the glenoid (0.4%; N = 1; Table 2).

**Table 2.** Summary of complications.

Author	Complications
Elamo et al [12]	13 recurrent instability of which 9 underwent re-revision with open Latarjet
	19 postoperative osteoarthritis
Slaven et al [42]	19 recurrent instability
Buckup et al [6]	3 re-dislocation
	2 persistent apprehension
Bartl et al [3]	4 recurrent instability
Ryu and Ryu [40]	1 recurrent instability
Barnes et al [2]	1 persistent apprehension
Neri et al [35]	2 re-dislocation
Stein et al [44]	1 re-dislocation
	4 persistent apprehension
	1 traumatic failure of the glenoid
Frank et al [16]	12 recurrent instability
Franceschi et al [15]	1 recurrent instability

One study (N = 53) found that those with postoperative instability had a significantly shorter (*P* = .029) durability of the index procedure compared to those who had a successful revision surgery (38.1 ± 31.3 months vs 20.5 ± 17.8 months) [42].

One study stratified recurrent instability rates for those who underwent revision arthroscopic Bankart repair after a failed primary arthroscopic Bankart repair, but did not stratify complications (eg, shoulder stiffness, revision for loose titanium anchor) for this population [3].

None of the included studies reported the use of remplissage to treat Hill-Sachs lesions. Of the 4 studies that reported the use of a rotator interval closure (RIC) [3,15,20,35], only 1 (N = 10) appropriately documented its distribution for patients undergoing revision arthroscopic Bankart repair after a failed index arthroscopic repair [15]. Of these patients, 70% (N = 7) had a RIC during revision arthroscopic Bankart repair. Patient positioning was not reported (57.7%; N = 162), lateral decubitus (28.5%; N = 80), or beach-chair (13.9%; N = 39). Of the studies that reported a mean of anchors used, 1 study (3.9%; N = 11) reported a mean of 2.5 anchors [40]; 1 study (3.6%; N = 10) reported a mean of 2.7 (range: 2-3) anchors [15]; 1 study (11.4%; n = 32) reported a range of 3 to 5 anchors used [3]; 1 study (2.1%; N = 6) reported a range of 3 to 6 anchors used [35]; and 1 study (2.5%; N = 7) reported a minimum of 3 anchors [20].

A total of 7 studies (N = 109) reported positioning of the anchors [3,6,15,20,35,40,44]. Of these, 2 studies (15.3%; N = 43) placed the most inferior anchor at the 4:30 o’clock position and the superior anchor at the 3:00 o’clock position [6,44]. Another 2 studies (13.9%; N = 39) placed the most inferior anchor at 5:30 o’clock [3,20]. One of these studies (2.5%; N= 7) specified that the remaining anchors were placed in the 4:30 and 3:00 o’clock positions [20]. One study (3.9%, N = 11) reported that the anchors were placed as close as possible to the glenoid surface without detracting the surface area in cases of glenoid bone loss [40]. Finally, 2 studies (5.7%; N = 16) placed the most inferior anchor at the 5:00 o’clock position [15,35].

There was considerable variation among included studies for indications on performing revision arthroscopic Bankart repair after failed index arthroscopic soft-tissue stabilization. All shoulders (N = 281) had recurrent instability after the index stabilization procedure. One study (3.6%; N = 10) selected patients for revision arthroscopic Bankart repair if persistent pain was present due to anterior inferior glenoid humeral instability that was non-responsive to a program of at least 6 months of non-operative treatment, consisting of avoidance of painful activities, use of non-steroidal anti-inflammatory medication, and participation in a physical therapy program designed to maintain or improve the strength in the shoulder girdle [15].

Three studies (38.4%; N = 108) reported that a revision arthroscopic Bankart repair was abandoned if there was a preoperative glenoid bone loss of > 20% [3,42,44]. One of these studies (11.5%; N = 32) also mentioned that a revision arthroscopic procedure was abandoned if there was a large Hill-Sachs defect in abduction and external rotation [3]. Another study (7.1%; N = 20) stated that revision arthroscopic Bankart repair was abandoned on shoulders with bony glenoid defects greater than 20%, a significant Hill-Sachs lesion or hyperlaxity, any concomitant abnormality of the biceps or the rotator cuff, and any osteoarthritic alterations of the joint [6]. One study (32.4%; N = 91), which compared the results between arthroscopic Bankart repair and Latarjet, performed Latarjet on those who had a glenoid bone loss of at least 25% [16].

Overall, 4 studies (N = 55) reported the use of an RIC supplemented with the revision arthroscopic Bankart repair [3,15,20,35). One study (N = 10) performed an arthroscopic RIC on patients (N = 7) who had persistent significant capsular laxity even after the repair of the capsulolabral complex [15]. The remaining 3 studies, comprised mixed populations, did not specify the distribution of RIC based on index stabilization procedure (ie, open versus arthroscopic repair), and reported the following indications for its use: (1) clinical signs of hyperlaxity or significant capsular laxity after the capsulolabral repair [3]; hyperlaxity (ie, positive sulcus sign persisting in external rotation) [20]; or residual capsular laxity, redundant rotator intervals, or a sulcus sign, or both, that did not resolve with external rotation of the extremity (Table 3) [35].

**Table 3.** Indications/contraindications of revision arthroscopic Bankart repair and adjunct procedures.

Author	No. of patients	Indications/Contraindications							
		Recurrent instability after index procedure	Persistent pain	Glenoid bone loss < 20%	Glenoid bone loss < 20%, significant Hill-Sachs lesion or hyperlaxity, any concomitant abnormality of the biceps or the rotator cuff, and any osteoarthritic alterations of the joint	Glenoid bone loss < 25%	RIC—persistent capsular laxity after repair of capsulolabral complex	RIC—Positive Sulcus Sign Persisting in External Rotation	RIC—Capsular laxity, redundant rotator interval or sulcus sign, or both, that did not resolve with external rotation of the extremity
Elamo et al [12]	30	x							
Buckup et al [6]	20	x			x				
Barnes et al [2]	9	x							
Stein et al [44]	23	x		x					
Ryu and Ryu [40]	11	x							
Frank et al [16]	62	x				x			
Franchesci et al [15]	10	x	X				x		
Slaven et al [42]	53	x		x					
Gill et al [18]	16	x							
Bartl et al [3]	32	x		x			x		
Imhoff et al [20]	7	x						X	
Neri et al [35]	6	x							x

*RIC* Rotator interval closure.

Shoulders were immobilized for 4 weeks (22.8%; N = 64), 6 weeks (11.4%; N = 32), 4 to 6 weeks (3.6%; N = 10), or 3 weeks (2.1%; N = 6); immobilization period was not specified for 60.1% (N = 169) of shoulders undergoing revision arthroscopic Bankart repair [16,18,20,42]. Of the included studies that reported return to sport or unrestricted activities, it was permitted at 6 months (18.9%; N = 53) [3,15,40], 10 months (15.3%; N = 43) [44], and 12 months (2.1%; N = 6) [35].

Two studies compared primary arthroscopic Bankart repair with revision arthroscopic Bankart repair [18,44]. One study found significant differences between the 2 groups, favoring the primary Bankart repair group, for the Rowe score (*P* = .001), Watch-Duplay score (*P* = .002), Constant score (*P* = .042), numerous analogue scale for pain (*P* = .023), high external rotation deficit (*P* = .001) and low external rotation deficit (*P* = .001) [44].

Two studies compared revision arthroscopic Bankart repair and open Latarjet [12,16]. One study found that patients undergoing revision arthroscopic soft-tissue Bankart repair had significant improvements postoperatively in American Shoulder and Elbow Surgeons (ASES), Shoulder Simple Test (SST), and Visual Analog Scale (VAS) scores (*P* < 0.001) [16]. Furthermore, the number of prior surgeries (*P* < .001) and baseline hyperlaxity (*P* = .04) were found to be significant risk factors for recurrent instability in revision arthroscopic patients. This study reported no direct comparisons between the arthroscopic and Latarjet groups [16]. Another study found significant differences favoring the Latarjet group in recurrent instability (*P* = .0007), Subjective Shoulder Value (*P* = .0368), Western Ontario Shoulder Instability index (*P* = .0166) and osteoarthritis at follow-up (*P* = .0318) [12].

Only 1 study reported data regarding sporting activity for patients undergoing revision arthroscopic Bankart repair after a failed primary arthroscopic repair [6]. A significant (*P* < .0001) postoperative reduction was found in subjective patient outcome for return to sports compared to before the first-time dislocation. Furthermore, 70% of patients returned to pre-injury level of sport. However, 90% had persistent deficits and shoulder-related limitations during sporting activity.

## Discussion

The most significant finding of this systematic review was that there exists a role for revision arthroscopic Bankart repair in patients after a failed index arthroscopic soft-tissue repair if there is minimal glenoid bone loss. After over 4 years of follow-up, patients without significant glenoid bone loss undergoing revision anterior arthroscopic Bankart repair had relatively moderate postoperative recurrent instability rates (19.9%), which is comparable to more standard techniques of revision such as open Bankart repair or the Latarjet procedure [9,14]. Furthermore, included studies reported significant improvements from preoperative to postoperative in patient-reported outcomes (eg, VAS, ASES scores). Finally, these results should be taken with caution due to the limited literature available on the topic, quality of studies, overall small sample size, and poor documentation of data across included studies. Henceforth, the *a priori* hypotheses were confirmed in this systematic review.

Of the several factors contributing to the success of revision arthroscopic Bankart repair, patient selection is pivotal [33,46]. Five of the included studies performed revision arthroscopic Bankart repair on patients who did not have substantial glenoid bone loss (ie, <20% or <25%) [3,6,16,42,44]. Patients with larger amounts of bone loss and engaging Hill-Sachs lesions are at risk of a failed arthroscopic Bankart repair and may be better managed with a bone transfer procedure [11,33,38,46]. None of the included studies reported the use of a remplissage to treat Hill-Sachs lesion. In fact, 2 studies excluded patients who had a significant Hill-Sachs lesion (ie, lesion of the subchondral bone) [3,6]. Surgeons should also consider the surgical cause of the failure of the index procedure such as anchor placement, and ensure that this cause is addressed in the revision repair [2]. Unfortunately, none of the studies included in our review reported on anchor placement in the prior arthroscopic Bankart repair so it is difficult to ascertain its influence on revision arthroscopic Bankart repair [2]. Furthermore, it is also important to appropriately evaluate and re-tension the capsulolabral tissue to prevent external rotation limitations, subsequent long-term glenohumeral osteoarthritis, and recurrent instability [7,8,21]. Adequate retensioning of the capsulolabral complex via intensive mobilization and elimination of the capsular pouch by a sufficient capsular shift and plication of tissue to prevent excessive capsule volume is also important for successful revision arthroscopic Bankart repair [19]. Surgeons should also assess shoulder laxity, since hyperlaxity of the shoulder can compromise a revision arthroscopic Bankart repair [33,46]. The use of a RIC or inferior plications can reduce capsular volumes in cases of hyperlaxity [10,17,20]. In the current systematic review, 4 studies reported the use of a RIC on patients who displayed hyperlaxity and/or had a persistent redundant capsule even after tightening [3,15,20,35]. Only 1 of these studies reported the distribution based on index stabilization procedure, of which 70% (7/10) of patients had an RIC during revision arthroscopic Bankart repair after a failed index arthroscopic soft-tissue procedure [15]. Next, in the current systematic review, the mean age of patients was 26.1 ± 3.8 years, indicating a very young cohort. With a lack of reporting across included studies on the influence of age and/or activity level on postoperative outcomes and complications, it is difficult to ascertain the influence of these factors on the success of revision arthroscopic Bankart repair.

Other factors contributing to a successful repair include technical proficiency in arthroscopic repair, positioning or number of anchors, and appropriate healing and rehabilitation [3,4,15,19,29,38,45]. Studies have reported that the use of fewer than 3 or 4 anchors increases the likelihood of recurrent instability [4,24,38], and it is recommended to use at least 4 anchor points to ensure proper stabilization, irrespective of the extent of the Bankart lesion [4]. Furthermore, the use of knotless anchors can also increase the risk of recurrent instability [38]. In the current systematic review, 16% of patients had at least 3 anchors and a maximum of 6, whereas 3.9% and 3.6% had a mean of 2.5 and 2.7 (range: 2-3) anchors, respectively. It is possible that the variation in the number of anchors used across the studies influenced the rate of recurrent instability (19.9%). Finally, return to unrestricted activities is recommended after proper healing and rehabilitation, which requires approximately 6 to 9 months based on the quality of tissue and repair [19]. In the current systematic review, patients returned to sport at either 6 months (18.9%), 10 months (15.3%), or 12 months (2.1%). With some patients returning to unrestricted activity earlier than recommended, the risk of recurrent instability can increase.

Two of the included studies compared primary vs revision arthroscopic Bankart repair [18,44]. One study found that revision patients had an expected minor decrease in the glenoid articulation arc parameters for the anterior portion [44]. The authors of this study suggest that this decrease, along with a reduction in the elasticity of the anterior capsule tissue and increased contact pressure load, may have contributed to the functional deficits and higher pain status that revision repair patients experienced [44].

Several studies have investigated the outcomes of revision anterior shoulder stabilization. A recent review found that revision anterior shoulder stabilization techniques such as arthroscopic, open Bankart, or bony procedures yielded satisfactory outcomes, with the lowest recurrent instability rate for patients undergoing the Latarjet procedure [28]. Another review found a complication and recurrence rate of 18.0% and 15.3%, respectively, for patients undergoing arthroscopic revision Bankart repair [50]. The authors noted that postoperative improvements are found if patients are selected carefully (ie, minimal glenoid bone loss and Hill-Sachs lesion) [50]. A systematic review published in 2013 found that arthroscopic revision Bankart repairs yield similar results to that of revision open Bankart repair [1]. These reviews, however, included studies of mixed populations of patients who had a failed index open or arthroscopic procedure. In our systematic review, all of the included patients underwent revision arthroscopic Bankart repair after a failed index arthroscopic Bankart repair. Given the increasing interest in the use of arthroscopy for anterior instability in both the primary and revision setting, it is important to evaluate its utility in isolation. However, in the current review, all included studies were of level III and level IV evidence, with a mean sample size of 23.3 (range: 6-62), highlighting the paucity of the available research on this novel topic and a dire need for better quality studies with large sample sizes.

Strengths stem from the thorough methodology employed in this review. A broad search strategy on multiple large databases ensured that no relevant studies were missed. The systematic screening approach was employed in duplicate, minimizing reviewer bias. Moreover, agreement among the 2 reviewers at all screening stages and quality assessment were excellent. This systematic review had an overall long-term follow-up (~5 years). With varying surgical techniques across included studies, the results of this systematic review are generalizable. Finally, this topic is of novel and increasing interest in orthopedics given the increase in sporting activity among the young, active population and the susceptibility of recurrent instability following an index arthroscopic Bankart repair.

The limitations of this systematic review stem from the quality of the available evidence, as all studies were of level III and level IV. The statistical and methodological heterogeneity among included studies precluded a meta-analysis. Instances of this heterogeneity include the study design, comparative groups, populations investigated, and follow-up periods. Furthermore, poor documentation of data (eg, primary repair details, revision surgical techniques and outcomes) limited our ability to ascertain the influence of adjunct procedures and determine accurate, patient-reported outcomes and return-to-sport rates. Studies did not document humeral sided or bipolar bone loss, limiting our ability to ascertain the influence of bony morphology on failure rates. Many studies did not specify whether recurrent instability/failure was defined as subluxation or dislocation, limiting the ability to appropriately assess the extent to which patients experienced failures after revision arthroscopic Bankart repair. Finally, an optimal soft-tissue revision Bankart repair technique following a failed primary arthroscopic Bankart repair is difficult to ascertain with the high heterogeneity of surgical techniques across included studies.

Future studies should use large prospective cohorts and long-term follow-up to determine more accurate failure and complication rates. These studies should also improve the documentation of all data (ie, demographic, outcomes, failures, revisions, complications, etc.) by index procedure (eg, open versus arthroscopic), as well as instability type (eg, anterior versus multidirectional). Future studies should seek to standardize the revision arthroscopic Bankart procedure (eg, patient positioning, number of anchors, anchor positioning, etc) and the rehabilitation protocol based on patient factors and clinical history to better guide clinicians on treating this patient population. Studies should determine the influence of adjunct procedures such as a remplissage or RIC on failure rates in comparison to arthroscopic revision Bankart repair performed in isolation.

In conclusion, with patients having significant improvement postoperatively and comparable recurrent instability rates, there exists a potential role in the use of revision arthroscopic Bankart repair after a failed primary arthroscopic Bankart repair where the glenoid bone loss is <20% to 25%. Clinicians should consider patient history and imaging findings to determine whether a more rigorous stabilization procedure is warranted. Future studies using large prospective cohorts and long-term follow-up and improved documentation of data are required to determine more accurate failure and complication rates.

## Supplemental Material

sj-docx-1-hss-10.1177_15563316211030606 – Supplemental material for Revision Arthroscopic Bankart Repair for Anterior Shoulder Instability After a Failed Arthroscopic Soft-Tissue Repair Yields Comparable Failure Rates to Primary Bankart Repair: A Systematic Review Click here for additional data file. Supplemental material, sj-docx-1-hss-10.1177_15563316211030606 for Revision Arthroscopic Bankart Repair for Anterior Shoulder Instability After a Failed Arthroscopic Soft-Tissue Repair Yields Comparable Failure Rates to Primary Bankart Repair: A Systematic Review by Ajaykumar Shanmugaraj, Seaher Sakha, Tushar Tejpal, Timothy Leroux, Jacob M Kirsch and Moin Khan in HSS Journal®: The Musculoskeletal Journal of Hospital for Special Surgery

sj-docx-2-hss-10.1177_15563316211030606 – Supplemental material for Revision Arthroscopic Bankart Repair for Anterior Shoulder Instability After a Failed Arthroscopic Soft-Tissue Repair Yields Comparable Failure Rates to Primary Bankart Repair: A Systematic Review Click here for additional data file. Supplemental material, sj-docx-2-hss-10.1177_15563316211030606 for Revision Arthroscopic Bankart Repair for Anterior Shoulder Instability After a Failed Arthroscopic Soft-Tissue Repair Yields Comparable Failure Rates to Primary Bankart Repair: A Systematic Review by Ajaykumar Shanmugaraj, Seaher Sakha, Tushar Tejpal, Timothy Leroux, Jacob M Kirsch and Moin Khan in HSS Journal®: The Musculoskeletal Journal of Hospital for Special Surgery
