# Supplementary material for: Revision Arthroscopic Bankart Repair for Anterior Shoulder Instability After a Failed Arthroscopic Soft-Tissue Repair Yields Comparable Failure Rates to Primary Bankart Repair: A Systematic Review
Source: HSS J. 2021 Jul 23;18(1):145–55. doi: 10.1177/15563316211030606 (PMC8753542; doi:10.1177/15563316211030606)
Supplement: sj-docx-2-hss-10.1177_15563316211030606 – Supplemental material for Revision Arthroscopic Bankart Repair for Anterior Shoulder Instability After a Failed Arthroscopic Soft-Tissue Repair Yields Comparable Failure Rates to Primary Bankart Repair: A Systematic Review [file sj-docx-2-hss-10.1177_15563316211030606.docx]

Appendix Table 1. Search strategy

| **EMBASE:** 1246 studies | **MEDLINE:** 816 studies | **PUBMED:** 89 studies |
| --- | --- | --- |
| **Strategy:**  1. Shoulder*.mp. or exp Shoulder Joint/ or exp Shoulder/  2. Glenohumeral.mp.  3. Capsul*.mp.  4. soft tissue.mp.  5. 1 or 2 or 3 or 4  6. sublux*.mp.  7. exp Shoulder Dislocation/ or dislocation.mp.  8. exp Joint Instability/ or instabil*.mp.  9. 6 or 7 or 8  10. revis*.mp.  11. exp Reoperation/ or reoperat*.mp.  12. fail*.mp.  13. 10 or 11 or 12  14. anterior.mp.  15. latarjet.mp.  16. exp Arthroscopy/ or arthroscop*.mp.  17. surg*.mp.  18. stabil*.mp.  19. repair*.mp.  20. remplissage.mp.  21. exp Bankart Lesions/ or bankart.mp.  22. 15 or 16 or 17 or 18 or 19 or 20 or 21  23. 5 and 9 and 13 and 14 and 22  24. limit 23 to (English language and humans) | **Strategy:**  1. Shoulder*.mp. or exp Shoulder Joint/ or exp Shoulder/  2. Glenohumeral.mp.  3. Capsul*.mp.  4. soft tissue.mp.  5. 1 or 2 or 3 or 4  6. sublux*.mp.  7. exp Shoulder Dislocation/ or dislocation.mp.  8. exp Joint Instability/ or instabil*.mp.  9. 6 or 7 or 8  10. revis*.mp.  11. exp Reoperation/ or reoperat*.mp.  12. fail*.mp.  13. 10 or 11 or 12  14. anterior.mp.  15. latarjet.mp.  16. exp Arthroscopy/ or arthroscop*.mp.  17. surg*.mp.  18. stabil*.mp.  19. repair*.mp.  20. remplissage.mp.  21. exp Bankart Lesions/ or bankart.mp.  22. 15 or 16 or 17 or 18 or 19 or 20 or 21  23. 5 and 9 and 13 and 14 and 22  24. limit 23 to (English language and humans) | **Strategy:**  Search ((((Latarjet OR Arthroscop* OR Surg* OR Stabil* OR Repair* OR Remplissage OR Bankart*)))) AND Anterior) AND (((Revis* OR Reoperat* OR Fail*)))) AND (((Sublux* OR Dislocation OR Shoulder Dislocation OR Shoulder Instability OR Instabil* OR Joint Instability)))) AND (((Shoulder* OR Shoulder Joint OR Glenohumeral OR Capsul* OR Soft Tissue))))) AND ("2019/04/29"[Date - Publication] : "3000"[Date - Publication]))))) |
